# A comparison of hemodynamic measurement methods during orthotopic liver transplantation: evaluating agreement and trending ability of PiCCO versus pulmonary artery catheter techniques

**DOI:** 10.1186/s12871-024-02582-x

**Published:** 2024-06-06

**Authors:** Yulu Feng, Zexi Ye, Yuekun Shen, Wei Xiong, Xiaoxiang Chen, Xiaoliang Gan, Shihong Wen, Lu Yang

**Affiliations:** 1 Departments of Anesthesiology, The First Affiliated Hospital, Sun Yat-sen University, Guangzhou, China; 2 Departments of Anesthesiology, Zhongshan Ophthalmic Center, Sun Yat-sen University, Guangzhou, China

**Keywords:** Liver transplantation, Hemodynamic monitoring, PiCCO, Pulmonary artery catheterization, Agreement analysis, Trending ability.

## Abstract

**Background:**

Significant hemodynamic changes occur during liver transplantation, emphasizing the importance of precious and continuous monitoring of cardiac output, cardiac index, and other parameters. Although the monitoring of cardiac output by pulse indicator continuous cardiac output (PiCCO) was statistically homogeneous compared to the clinical gold standard pulmonary artery catheterization (PAC) in previous studies of liver transplantation, there are fewer statistical methods for the assessment of its conclusions, and a lack of comparisons of other hemodynamic parameters (e.g., SVRI, systemic vascular resistance index). Some studies have also concluded that the agreement between PiCCO and PAC is not good enough. Overall, there are no uniform conclusions regarding the agreement between PiCCO and PAC in previous studies. This study evaluates the agreement and trending ability of relevant hemodynamic parameters obtained with PiCCO compared to the clinical gold standard PAC from multiple perspectives, employing various statistical methods.

**Methods:**

Fifty-two liver transplantation patients were included. Cardiac output (CO), cardiac index (CI), SVRI and stroke volume index (SVI) values were monitored at eight time points using both PiCCO and PAC. The results were analyzed by Bland-Altman analysis, Passing-bablok regression, intra-class correlation coefficient (ICC), 4-quadrant plot, polar plot, and trend interchangeability method (TIM).

**Results:**

The Bland-Altman analysis revealed high percentage errors for PiCCO: 54.06% for CO, 52.70% for CI, 62.18% for SVRI, and 51.97% for SVI, indicating poor accuracy. While Passing-Bablok plots showed favorable agreement for SVRI overall and during various phases, the agreement for other parameters was less satisfactory. The ICC results confirmed good overall agreement between the two devices across most parameters, except for SVRI during the new liver phase, which showed poor agreement. Additionally, four-quadrant and polar plot analyses indicated that all agreement rate values fell below the clinically acceptable threshold of over 90%, and all angular deviation values exceeded ± 5°, demonstrating that PiCCO is unable to meet the acceptable trends. Using the TIM, the interchangeability rates were found to be quite low: 20% for CO and CI, 16% for SVRI, and 13% for SVI.

**Conclusions:**

Our study revealed notable disparities in absolute values of CO, CI, SVRI and SVI between PiCCO and PAC in intraoperative liver transplant settings, notably during the neohepatic phase where errors were particularly pronounced. Consequently, these findings highlight the need for careful consideration of PiCCO’s advantages and disadvantages in liver transplantation scenarios, including its multiple parameters (such as the encompassing extravascular lung water index), against its limited correlation with PAC.

**Supplementary Information:**

The online version contains supplementary material available at 10.1186/s12871-024-02582-x.

## Background

Organ transplantation has revolutionized the field of medicine, with orthotopic liver transplantation(OLT) being the most effective treatment for end-stage liver disease and acute liver failure over the past few decades. Continuous and precise hemodynamic monitoring is essential to prevent and treat potentially serious adverse events during the perioperative period, especially intraoperatively, and to ensure tissue perfusion and postoperative recovery, which is an important guarantee of successful liver transplantation [1, 2].

Patients with liver failure often suffer from abnormal coagulation function or even acute hemorrhagic coagulation dysfunction due to coagulation factor deficiencies, thrombocytopenia, etc. Bleeding is the most common clinical manifestation, and large amounts of bleeding can cause hemodynamic fluctuations or even shock [3–6]. In addition, intraoperative hemodynamic fluctuations during liver transplantation are also common due to pre-existing hepatic insufficiency, high-output low-resistance states, reperfusion injury, surgery type, anesthesia, and patient positioning. Therefore, the accurate and continuous monitoring of cardiac output (CO), cardiac index (CI), systemic vascular resistance index (SVRI), and stroke volume index (SVI), which provide essential information for therapeutic interventions to ensure adequate tissue perfusion, is crucial for perioperative hemodynamic management. As described previously, thermodilution CO via a floating pulmonary artery catheter (PAC) is the accepted clinical gold standard; however, this method is expensive and complex, and the operation itself can cause many serious complications such as pneumothorax, arrhythmia, air embolism, and pulmonary artery rupture [7–9]. Furthermore, some studies have suggested that PAC monitoring failed to benefit critically ill patients [10]. Therefore, less invasive and safer methods that could accurately monitor hemodynamic parameters in the surgical and intensive care settings remain to be developed [11].

The use of Pulse integral Continuous Cardiac Output (PiCCO) in critically ill patients is increasing as it provides immediate volume parameters through transpulmonary thermodilution calibration and automatically calibrates arterial pulse curve analysis to provide a continuous parameter [12]. Meanwhile, it is a minimally invasive technique that requires only arterial and venous access [13] and has been found to correlate well with CO measured simultaneously using PAC in many basic and clinical studies [14–16]. However, one meta-analysis concluded that there was high heterogeneity among clinical studies comparing PiCCO and PAC in adult surgical and critical patients [17]. In liver transplantation, it has been argued that intermittent cardiac output monitoring compared with pulmonary artery catheterization has questionable ability to trend between the two techniques [18]. A previous study have shown a mean difference between PiCCO and PAC regarding cardiac output of 0.04 L min^− 1^ and a level of agreement of 1.69 L min^− 1^, indicating that PiCCO was accurate in liver transplantation [19]. However, there is no mention of whether the two agree regarding other hemodynamic parameters (e.g., SVRI). Therefore, a comprehensive assessment and uniform conclusion regarding the agreement and follow-up ability of PiCCO for hemodynamic monitoring is still lacking.

In the current study, we aim to evaluate the agreement and trending ability of hemodynamic monitoring using the PiCCO technique with pulse contour in orthotopic liver transplantation by comparing it with the PAC techniques.

## Methods

This comparative study was conducted at the First Affiliated Hospital of Sun Yat-sen University after receiving approval from the ethics committee (Approval number: 2017 − 310) and all patients provided written informed consent. Our data from previous studies of database has been registered in https://clinicaltrials.gov/ (ChiCTR-OPN-17,013,819). The intraoperative and post-transplant care was performed according to standard protocols applied at our center and a prior protocol publication [20]. The data were collected from the hospital database, and a total of 52 patients who underwent orthotopic liver transplantation were included in the analysis. The inclusion criteria were patients age over18 years who received both PAC and PiCCO monitoring during surgery. Patients with severe pulmonary disease, severe cardiac disease, and persistent arrhythmias were excluded from the study.

### Anesthesia and surgical protocol

Both anesthesia procedures and measurements were conducted by two experienced senior anesthesiologists. Patients were given general anesthesia with intravenous propofol (2 mg/kg), sufentanil (0.5 ug/kg), cis-atracurium (0.2 mg/kg) or rocuronium (0.9 mg/kg). Maintenance of anesthesia was achieved with a continuous infusion of propofol, remifentanil and sevoflurane inhalation. Mechanical ventilation was administered via endotracheal intubation under visual laryngoscopy, with a tidal volume of 6–10 mL/kg, respiratory rate of 10–20 breaths/min, and maintenance of end-expiratory carbon dioxide (PetCO2) between 35 and 45 mmHg. All intravenous fluids and blood products were warmed by a liquid warmer before administration. Dopamine, norepinephrine, or epinephrine were administered as need based on blood pressure, cardiac output, and central venous pressure parameters. A single intravenous administration of epinephrine and metaraminol was given if necessary. Red blood cells, fresh frozen plasma, and prothrombin complex were transfused as required based on the results of blood gas analysis and thromboelastography (TEG) during the surgery. A PiCCO pressure monitoring tube with an integrated temperature probe was placed in the left internal jugular vein, and the right femoral artery was punctured and connected to the PiCCO monitor (Pulsion Medical Systems, Munich, Germany). Concurrently, a floating catheter was carefully positioned within the right internal jugular vein and interfaced with a cardiac output monitoring system (7.5 F Swan-Ganz CCOmbo catheter, Edwards Lifesciences, Irvine, CA, United States). A PAC catheter was then connected to a Vigilance™ hemodynamic monitor (Edwards Lifesciences). Patients were transferred to ICU after surgery and kept on mechanical ventilation until complete recovery of spontaneous breathing and then the tracheal tube was removed.

### Data collection

Data was collected at eight distinct time points: T1 (abdominal incision), T2 (10 min before portal block), T3 (10 min after portal block), T4 (10 min before reperfusion), T5 (10 min after reperfusion), T6 (30 min after reperfusion), T7 (60 min after reperfusion) and T8 (abdominal closure). Simultaneous measurements including CO, CI, SVRI and SVI were conducted using both PiCCO (test technique) and PAC (reference technique) systems, following the established anesthesia and surgical protocol. Data measured by PiCCO and PAC are measured at the same point in time. During measurements, 15 mL of chilled normal saline was injected through the proximal lumen of the PAC and 20 mL saline through a central venous catheter at random phase of the respiratory cycle. At each time point, the median of value was calculated from five repeated measurements to serve as a representative value for trend analysis.

### Agreement analysis

Following the guidelines of the Clinical and Laboratory Standards Society NCCLS (2002), Bland-Altman analysis, the Passing-Bablok regression (PBR) and Intra-class Correlation Coefficient (ICC) were employed to evaluate the consistency between the parameter values obtained from the two methods. The results of the Bland-Altman analysis are shown as bias and 95% limit of agreement. The reference values for the CO, CI, SVI, and SVRI were calculated using values obtained from PAC. Percentage error (PE) was calculated using Critchley’s formula (1.96×standard deviation/mean); for PEs < 30%, PiCCO was considered interchangeable with PAC. [21, 22].

When comparing the new method with the current reference method, the two techniques are considered identical and commensurable if the confidence interval of the PBR coefficient (slope) b contains 1 and the confidence interval of the overall intercept a contains 0. If the confidence interval of the regression coefficient (slope) b does not contain 1, the two systems are considered to be proportionally different from each other. Similarly, if the confidence interval of the intercept does not contain 0, it signifies a systematic distinction between the two systems.

For ICC, a value greater than 0.75 means “excellent” agreement between the two methodologies, whereas a value less than 0.40 indicates a level of agreement characterized as “poor”. Intermediate values fall within the category of generally considered “good” agreement. A higher ICC value implies a reduced variance attributable to both systematic and random errors.

### Trending ability

According to the guidelines for reporting reliability and agreement studies, the assessment of agreement trends between the two methods involved the use of the four-quadrant plot, polar plot and Trend Interchangeability Method (TIM) [23, 24].

In studies comparing a single CO measurement, the four-quadrant plot and the polar plot were used to describe trending capabilities. Data at the center of the four-quadrant plot and the polar plot indicated minor variations in parameters, which were attributed to a considerable random error and considered a statistical noise component. An exclusion zone of 10% was implemented for percentage change data to eliminate such points. The radial agreement limits typically ranged from − 30 to + 30°. After excluding data from the central area, a concordance rate above 95% was considered as indicative of good trending ability, while rates falling between 90 and 95% was considered marginal, and those below 90% was deemed poor [25].

The TIM, in adherence to the GRRAS guidelines, is founded on the principle of classifying each change as either uninterpretable or interpretable, based on an analysis of the repeatability of the reference method. These changes are further classified as non-interchangeable, within a gray area, or interchangeable. The interchangeability rate, calculated as the number of interchangeable variations divided by the total number of interpretable variations, serves as a key metric. Its value is defined as excellent (≥ 95%), good (≥ 90%), poor (75-90%), or not clinically significant (< 75%) based on the interchangeability rate. All TIM analyses were performed using established methods, including R programs and Excel tables.

### Statistical analysis

Normality of data was assessed using the Shapiro-Wilk test. Accordingly, continuous data were expressed as mean ± SD or median (25% percentile, 75% percentile) of patients and compared with independent *t* test or Mann-Whitney *U* test, respectively. Categorical data were expressed as frequency or percentage. Deviation was defined as the mean difference observed between the two methods. Statistical analyses for assessing trending ability analysis were carried out using MedCalc (MedCalc statistical software CA, United States), IBM SPSS Statistics for Windows (version 20.0, IBM Corp, Armonk, NY, United States) and Microsoft Excel for Mac 2011 (version 14.4.7, Microsoft Corporation, Redmond, WA). *P* < .05 was considered statistically significant.

## Results

### Cohort characteristics

**Fig. 1:**
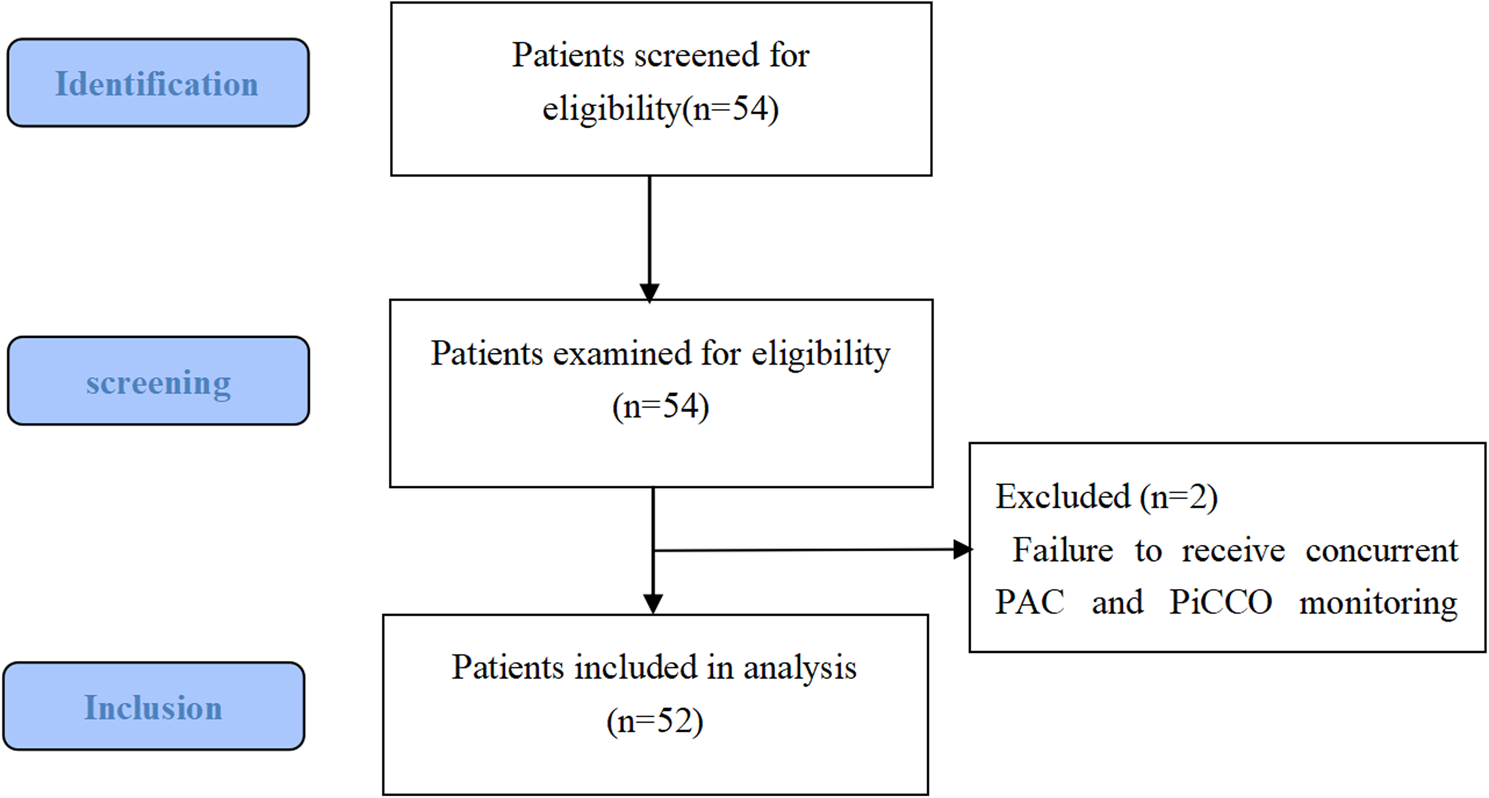
Shows eligible patients’ selection diagram

A total of 52 patients underwent OLT were included in the current study (Fig. 1). The clinical and surgical characteristics of the patients are listed in Table 1. None of the patients suffered from serious heart or valvular disease.

**Table 1:** Baseline patient characteristics (*n* = 52)

Characteristic	
Age(yr) (mean, range)	54(30–72)
Height(cm)	167(6)
Weight(kg)	67(12)
MELD-score	16[11–43]
LVEF(%)	71(5)
e/a	1.15(0.35)
Surgery time(min)	417(100)
Sex, (male/female) (n)	45/7
Underlying disease	
Cirrhosis HBV related [n (%)]	19(36)
Cirrhosis HCV related [n (%)]	1(2)
Alcoholic cirrhosis [n (%)]	2(4)
Primary biliary cirrhosis [n (%)]	1(2)
Autoimmune cirrhosis [n (%)]	1(2)
Acute Liver Failure [n (%)]	2(4)
Hepatocellular carcinoma [n (%)]	26(50)
Child classification(A/B/C)(n)	12/19/21
Type of surgery (classic procedure/backpack procedure/ischemia-free procedure)(n)	18/8/26

Continuous data are expressed as the mean (SD) or median [25th–75th percentile]. Categorical data were expressed as number (%)

Abbreviations: MELD, model for end-stage liver disease; LVEF, left ventricular ejection fraction; e, early maximal ventricular filling velocity; a, atrial maximal ventricular filling velocity; HBV, hepatitis B virus; HCV, hepatitis C virus

**Table 2:** Hemodynamic measurements at each time point

	T1	T2	T3	T4	T5	T6	T7	T8
HR(beats/min)	64 ± 11	82 ± 18^b^	86 ± 21^b^	82 ± 20^b^	82 ± 17^b^	84 ± 19^b^	83 ± 18^b^	75 ± 18^b^
MAP(mm Hg)	77 ± 12	77 ± 11	76 ± 12	70 ± 11^a^	67 ± 13	69 ± 13^a^	75 ± 11	75 ± 17
CVP(cm H2O)	10 ± 5	6 ± 4	5 ± 4	5 ± 3	7 ± 5	7 ± 4	8 ± 4	12 ± 9
PAC CO(L/min)	5.9(4.7−7)	5.4(4.2–6.6)	6.6(5.4–7.4)	5.6(4.7–6.8)	4.8(4.1–5.7)	6.1(5.0–7.0)	7.5(6.4–9.4)	7.3(6.5–8.6)
Pi CO(L/min)	6.1(5.0−7.1)	6.4(5.7−8.0)^d^	6.3(5.5–7.5)	6.1(5.2–6.7)	6.3(5.5–8.1)^d^	7.0(6.1–8.6)^d^	7.6(6.9–8.7)	7.3(6.5–8.5)
PAC CI(L/min/m^2)	3.4(2.8−4.0)	3.1(2.5–3.9)	3.6(3.0−4.4)	3.2(2.7–3.9)	2.8(2.3–3.4)	3.3(3.0−4.4)	4.4(3.7–5.8)	4.2(3.8–5.3)
Pi CI(L/min/m^2)	3.5(2.9–3.9)	3.8(3.2–4.4)^d^	3.7(3.2–4.5)	3.6(3.0−3.9)	3.7(3.1–4.5)^d^	4.2(3.5−5.0)^d^	4.3(4.1–5.3)	4.1(3.7−5.0)
PAC SVRI(dyn·s/cm^5/m^2)	1469(1173–1939)	1636(1313–2009)	1503(1305–1828)	1696.5(1469–2164)	1754.0(1506–2142)	1375.5(1170–1699)	1147(939–1415)	1245.0(1027–1478)
Pi SVRI(dyn·s/cm^5/m^2)	1580(1316–2202)	1289(1085–1658)^d^	1546.8(1265–1873)	1675.4(1292–2005)	1462.9(1182–1866)^d^	1250.9(1053–1501)^d^	1189.2(946–1367)	1335.6(1173–1592)^d^
PAC SVI(ml/m^2)	53 ± 13	41 ± 14	46 ± 13	42 ± 11	38 ± 14	45 ± 13	59 ± 15	57 ± 10
Pi SVI(ml/m^2)	54 ± 12	51 ± 14^d^	48 ± 15	45 ± 13	48 ± 15^d^	54 ± 15^d^	58 ± 15	54 ± 13^c^

a *P* < .05, b *P* < .01 versus T1

c *P* < .05, d *P* < .01 versus PAC

Continuous data are expressed as the mean (SD) or median [25th–75th percentile]. Abbreviations: HR, heart rate; MAP, mean arterial pressure; CVP, central venous pressure; PAC CO, cardiac output obtained by pulmonary artery catheter; Pi CO, cardiac output obtained by PiCCO; PAC CI, cardiac index obtained by ppulmonary artery catheter; Pi CI, cardiac index obtained by PiCCO; PAC SVRI, systemic vascular resistance index obtained by pulmonary artery catheter; Pi SVRI, systemic vascular resistance index obtained by PiCCO; PAC SVI, stroke volume index obtained by pulmonary artery catheter; Pi CO, stroke volume index obtained by PiCCO

Intraoperative CO, CI, SVRI, and SVI measurements at different time points are shown in Table 2. As shown, there were no significant differences in CVP over time, whereas all other parameters exhibited fluctuations. MAP showed a significant decrease at T6 compared to T1 (*P* < .05 for T6 versus T1). The values of the various parameters measured at T2, T5, T6, and T8 time points exhibited variations between the two device.

### Agreement was evaluated using bland-altman analysis

**Fig. 2:**
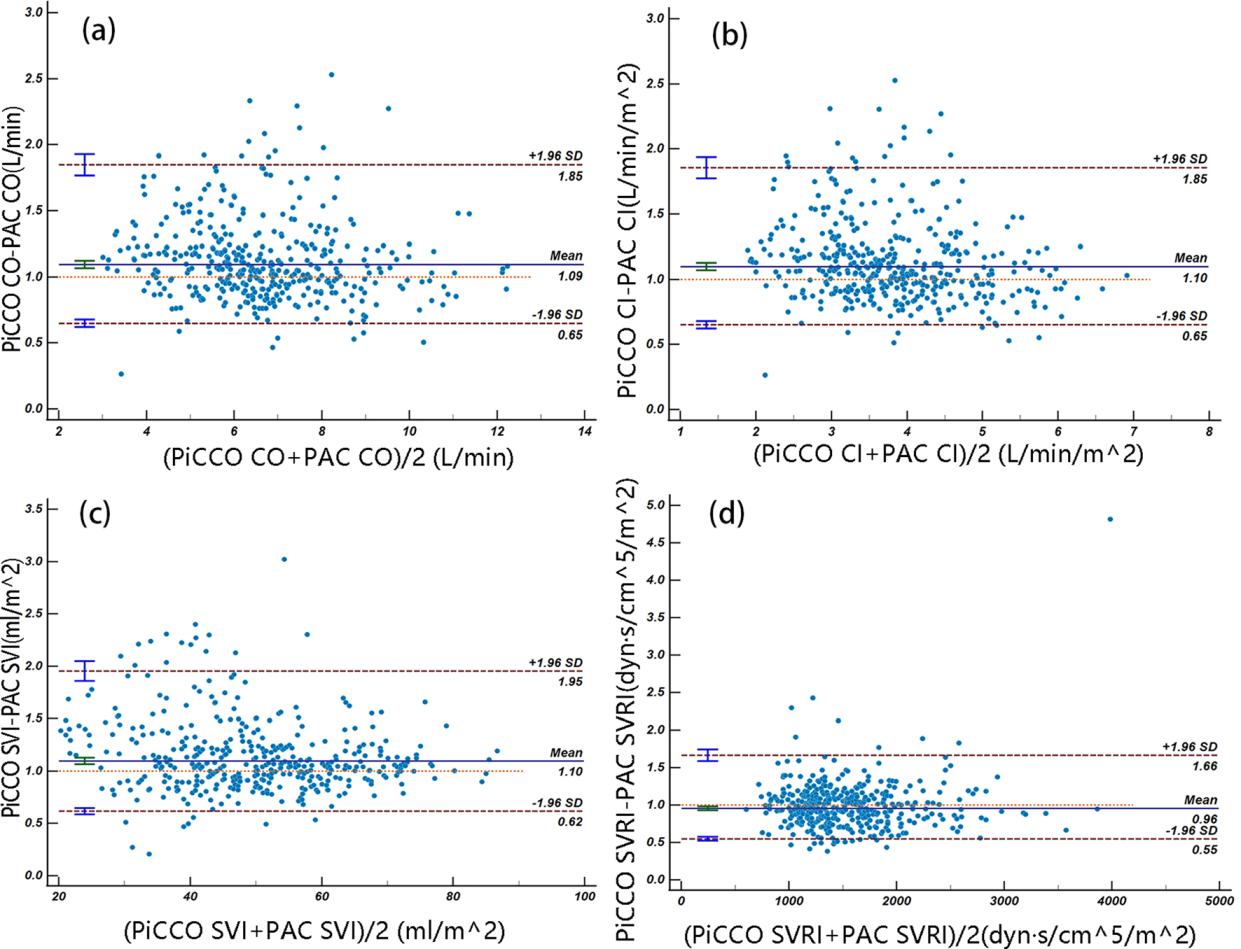
Agreement was assessed using Bland-Altman analysis. (**a**) Bland-Altman analysis comparing the CO measured using PiCCO with that using PAC. (**b**) Bland-Altman analysis comparing the CI measured using PiCCO with that using PAC. (**c**) Bland-Altman analysis comparing the SVI measured using PiCCO with that using PAC. (**d**) Bland-Altman analysis comparing the SVRI measured using PiCCO with that using PAC. The blue line indicates the mean bias, and the dashed lines indicate the 95% limits of agreement in each analysis. SD, standard deviation

The Bland-Altman analyses are shown in Fig. 2. The bias and 95% limits of agreement between the COs from PAC and those from PiCCO were 0.52 L/min and − 2.93 to 3.97. And those between the CIs were 0.30 min/ mL/m^2^ and − 1.63 to 2.23. And those between the SVIs were 3.99 mL/m^2^ and − 20.65 to 28.63. And those between the SVRIs were − 66.32 dyne.s/cm^5^/m^2^ and − 1049.16 to 916.52, respectively. The PEs of PiCCO were 54.06%, 52.70%, 51.97% and 62.18% for CO, CI, SVI and SVRI, respectively (Fig. 2), indicating poor accuracy.

### Agreement was evaluated using PBR

**Fig. 3:**
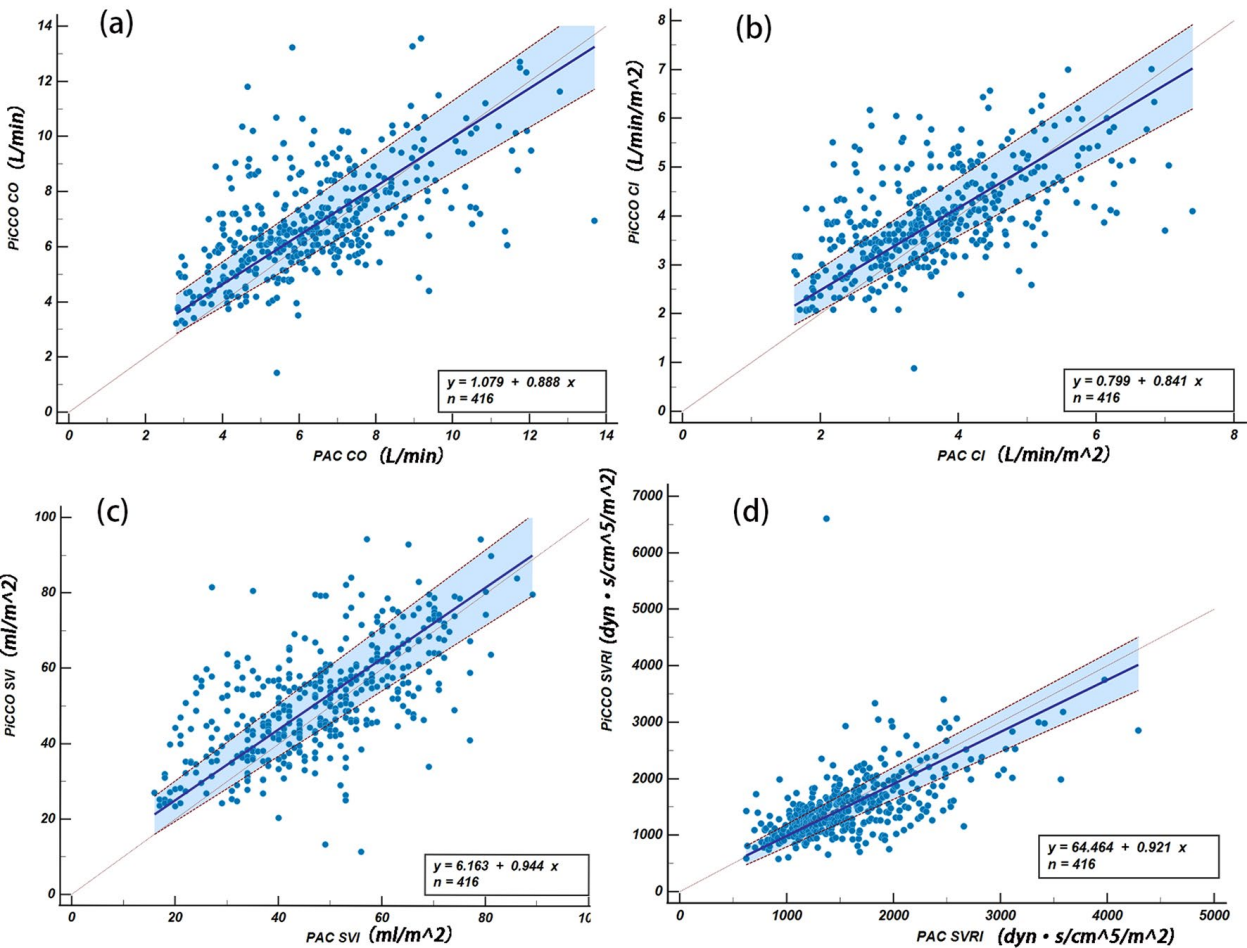
Agreement was assessed using Passing-Bablok regression (PBR). PBR between PiCCO and PAC for CO (**a**), for CI (**b**), for SVI (**c**), for SVRI (**d**)

Figure 3 shows the passing-bablok plots for each blood flow parameter measured by the two devices. The plots indicated a favorable agreement in overall and phase SVRI, but a less satisfactory agreement for the remaining parameters.

### Agreement was evaluated using ICC

For the model and definition of ICC, we choose a two-way random effects model with type absolute consistency. The ICC results indicated that there was good overall agreement between the two devices for all phases except for the SVRI in the new liver phase, where the devices demonstrated poor agreement (Table 3).

**Table 3:** ICC results for each phase between the two devices

	Co at all time points	Anhepatic prophase	anhepatic phase	Neohepatic phase
ICC	0.571	0.804	0.461	0.570
Agreement	good	excellent	good	good
	CI at all time points	Anhepatic prophase	anhepatic phase	Neohepatic phase
ICC	0.549	0.782	0.433	0.527
Agreement	good	excellent	good	good
	SVRI at all time points	Anhepatic prophase	anhepatic phase	Neohepatic phase
ICC	0.602	0.774	0.595	0.364
Agreement	good	excellent	good	Poor
	SVI at all time points	Anhepatic prophase	anhepatic phase	Neohepatic phase
ICC	0.611	0.630	0.542	0.596
Agreement	good	good	good	good

ICC > 0. 75 means excellent agreement between the two methods; ICC < 0. 40 means “poor” agreement; in between is generally considered good

### Trending ability assessment

**Fig. 4:**
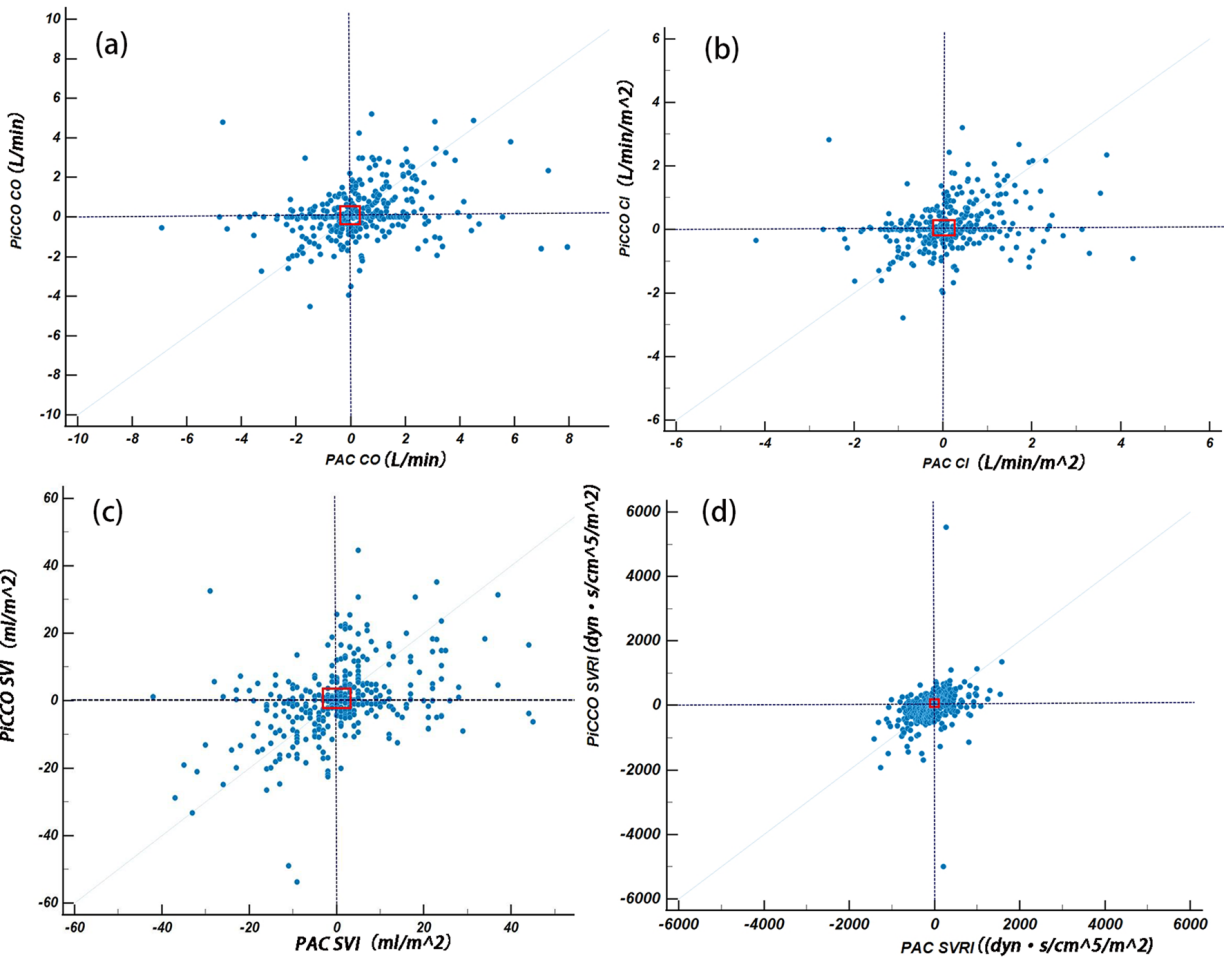
Trending ability was assessed using the four quadrant plots. Four-quadrant plot corrected for repeated measurements shows changes in CO(**a**), CI (**b**), SVI (**c**), SVRI (**d**). The exclusion rates (red squares) for the central region were both set to 10% of the parameter mean

**Fig. 5:**
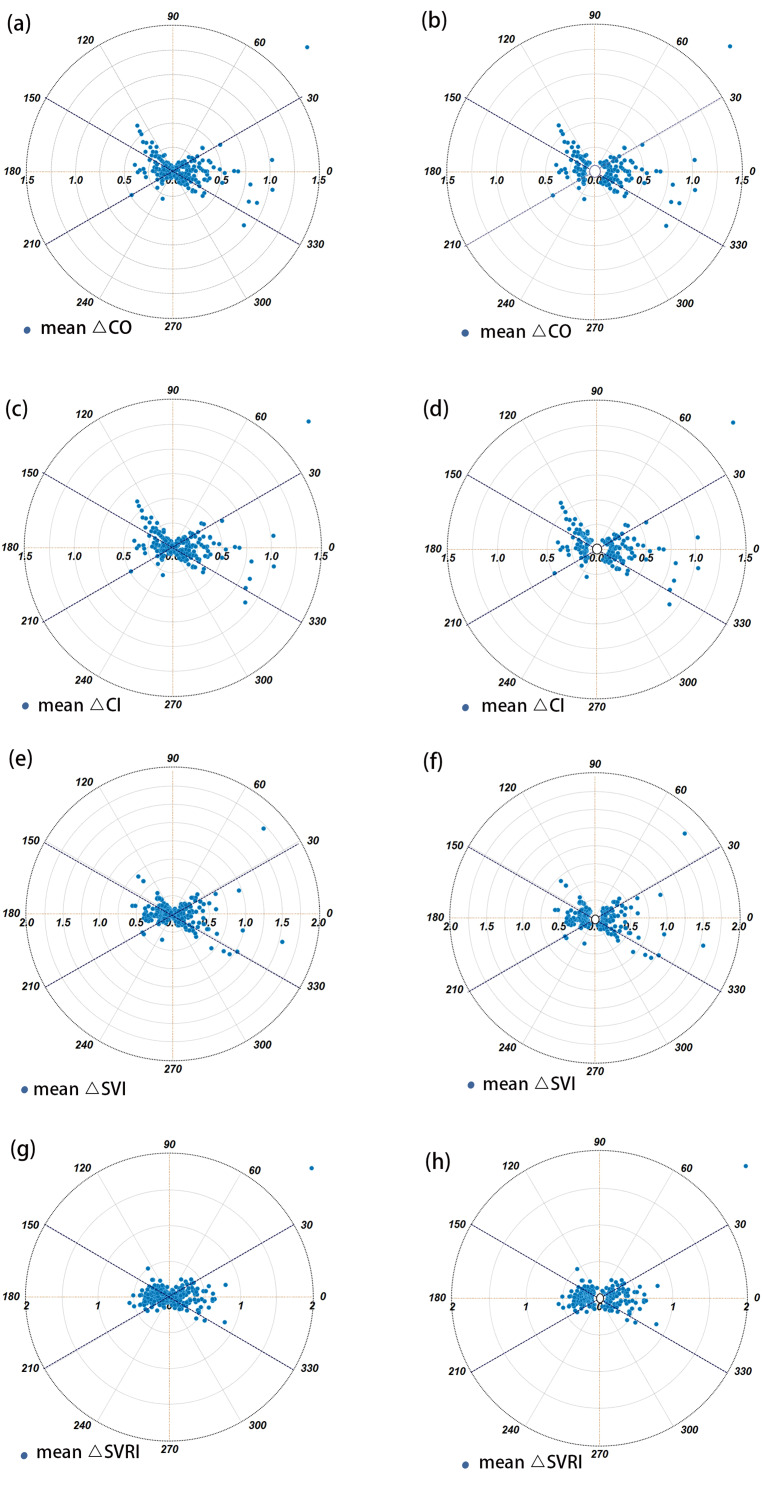
Trending ability was assessed using polar plot. The distance from the center of the plot represents the mean change in cardiac output (△CO) and the angle with the horizontal (0-degree radial) axis represents agreement (**a**), the exclusion zones of 10% (**b**). The distance from the center of the plot represents the mean change in CI (**c**), the exclusion zones of 10% (**d**). The distance from the center of the plot represents the mean change in SVI (**e**), the exclusion zones of 10% (**f**). The distance from the center of the plot represents the mean change in SVRI (**g**), the exclusion zones of 10%(**h**). The radial agreement limit was taken as -30 to + 30°, and after excluding data from the central area, a compliance rate above 95% was considered good trend ability, 90% ~ 95% was borderline, and below 90% was poor trend ability

After performing subtraction to create ΔCO、ΔCI、ΔSVRI、ΔSVI, a total of 364 data sets comparing PiCCO to PAC were available for trending analysis. The four-quadrant plots are shown in Fig. 4, and the polar plots are shown in Fig. 5. The exclusion rates for the central region were both set at 10% of the parameter mean. Following the exclusion of data from the central zone, the agreement rate and angular deviation between the two methods are presented in Table 3. The polar plot analysis showed that all agreement rate values were below the clinically acceptable standard of more than 90%, and all angular deviation values exceeded ± 5°, falling outside the bounds of acceptable trend capacity [25].

### TIM analysis

The TIM identified one change that was deemed not interpretable (indicated in blue, representing an overlap of confidence intervals). Among the interpretable changes, there was one point that was not interchangeable (highlighted in red, representing that neither the second point nor its repeatability fell within the interchangeability zone defined by the interchangeability lines). Additionally, there was one change within the gray zone (depicted in orange, where only the repeatability of the second point was within the interchangeability zone), and one change was deemed interchangeable (shown in green, with the second point falling within the interchangeability zone).

**Fig. 6:**
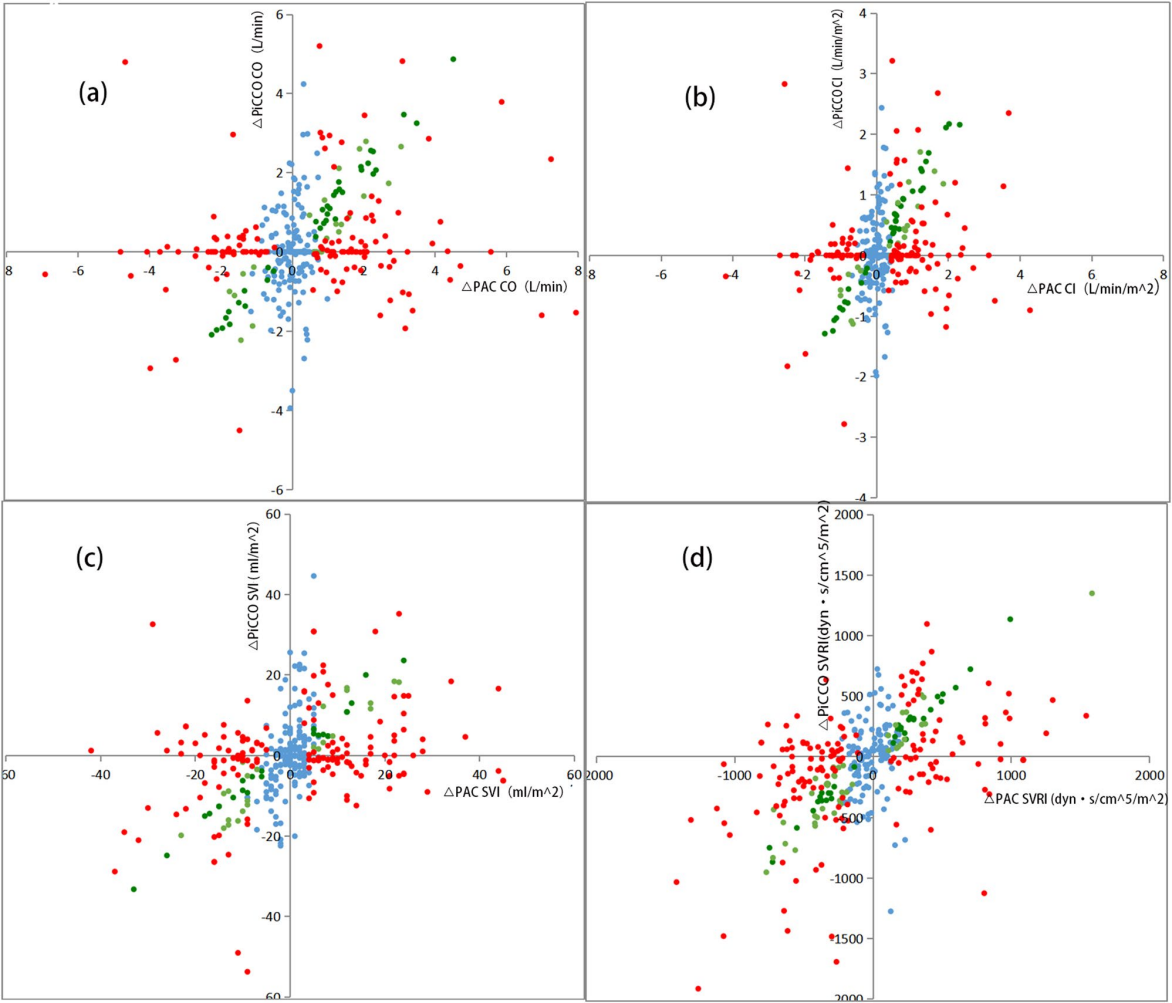
(**a**) Four-quadrant graphical representation between changes in absolute values of CO measured by PiCCO and PAC ( 364 pairs of data points) according to the trend interchangeability method(TIM). (**b**) Four-quadrant graphical representation between changes in absolute values of CI measured by PiCCO and PAC according to the TIM. (**c**) Four-quadrant graphical representation between changes in absolute values of SVI measured by PiCCO and PAC according to the TIM. (**d**) Four-quadrant graphical representation between changes in absolute values of SVRI measured by PiCCO and PAC according to the TIM. A specific colour is applied to each change: uninterpretable (blue), non-interchangeable (red), in the gray zone of interpretation (orange), and interchangeable (green)

Using the TIM, it was determined that 174 changes in cardiac output were uninterpretable. Among the remaining 190 (53%) interpretable changes, 37 (20%) were deemed interchangeable, 22 (11%) were within the gray zone, and 131(69%) were not interchangeable (Fig. 6). Using the same method, the interchangeability rates were calculated to be 20% for CI, 16% for SVRI, and 13% for SVI. The consistency rates for the three trend- tracking methods are summarized in Table 4.

**Table 4:** The consistency rates of the three methods of tracking trends

	Clinically acceptable standard	CO	CI	SVRI	SVI
Concordance rate of 4-quadrant plot(n%)	≥ 90	73	73	74	70
Angular deviation of polar plot(n°)	-5 ~ 5	72	72	95	85
Concordance rate of polar plot(n%)	≥ 90	48	49	68	56
The interchangeability rate of TIM(n%)	≥ 90	20	20	16	13

## Discussion

In our study, we assessed the consistency and trending ability of CO, CI, SVRI, and SVI measurements using PiCCO compared to PAC during OLT. The Bland-Altman analysis revealed high percentage errors for PiCCO: 54.06% for CO, 52.70% for CI, 62.18% for SVRI, and 51.97% for SVI, indicating poor accuracy. While Passing-Bablok plots showed favorable agreement for SVRI overall and during various phases, the agreement for other parameters was less satisfactory. The ICC results confirmed good overall agreement between the two devices across most parameters, except for SVRI during the new liver phase, which showed poor agreement. Additionally, four-quadrant and polar plot analyses indicated that all agreement rate values fell below the clinically acceptable threshold of over 90%, and all angular deviation values exceeded ± 5°, demonstrating that neither device reliably followed clinically acceptable trends. Using the TIM, the interchangeability rates were found to be quite low: 20% for CO and CI, 16% for SVRI, and 13% for SVI. These findings underscore that PiCCO does not compare favorably with PAC during OLT.

The patients undergoing liver transplant are subject to intense intraoperative hemodynamic fluctuations due to their pathophysiology and the specificity of the surgical procedure. Accurate and continuous monitoring of parameters such as cardiac output (CO), cardiac index (CI), systemic vascular resistance index (SVRI), and stroke volume index (SVI) is essential for perioperative hemodynamic management, and thermodilution of CO by floating pulmonary artery catheterization (PAC) is the accepted clinical gold standard. Nowadays, PAC are mainly used in patients having cardiac surgery, liver transplantation, and in critically ill patients with circulatory shock, especially with right ventricular dysfunction [12]. However, PAC is more invasive and is associated with various complications, including arrhythmias, pulmonary infarction, intrapulmonary hemorrhage, etc [26, 27], and due to coagulation function of liver transplantation patients and other pathophysiologic abnormalities, complications such as bleeding are more likely to occur.

Due to the high risk of PAC, technological advances in minimally invasive or noninvasive related monitoring devices are necessary. Several such alternatives have been developed, including transthoracic impedance methods, transesophageal echocardiography, arterial wave contour analysis and transpulmonary thermodilution [11, 28]. The latest research now concludes that PAC is the undisputed gold standard for hemodynamic monitoring in liver transplantation patients; however, it is highly invasive, and its use should be individualized. Cardiac output devices based on pulse contour analysis are minimally invasive devices with the advantage of real-time beat to beat monitoring of cardiac output [29]. PiCCO has gained widespread utilization, employing a combination of transpulmonary thermodilution and pulse contour analysis. Previous studies suggest its comparability to the PAC’s thermodilution method [30, 31], as well as less invasive and rare complications (e.g., inflammation and catheter-related infections) [26]. It also offers the advantage of the capacity to measure unique parameters, encompassing extravascular lung water index (EVLW), global end-diastolic volume (volume within the heart at end-diastole), and intrathoracic blood volume (volume within the heart and pulmonary circulation), all crucial for assessing cardiac load [28]. One such parameter, EVLW index, serves as an indicator of volume, cardiopulmonary function, and prognosis. It can intuitively reflect the severity of acute pulmonary edema, which may result from increased permeability and elevated hydrostatic pressure in the lungs. Continuous monitoring of EVLW is crucial for clinicians to accurately assess alveolar fluid volume, interstitial fluid volume, and gas diffusion function [32–34]. In our study, an EVLW greater than 10 warranted a high suspicion of pulmonary edema. By monitoring EVLW, we could effectively guide our interventions, including cardiac tonicity adjustments, diuretic therapy, ventilator settings optimization, and infusion regimen modifications. Thus, the unique parameters provided by PiCCO, such as the EVLW index and the pulmonary vascular permeability index, offer a robust basis for tailoring treatment.

However, opinions regarding the agreement between PICCO and PAC are differ among studies. Although the previous study in liver transplantation has been statistically homogeneous, it lack comparisons regarding other hemodynamic parameters, such as SVRI [18]. A comprehensive assessment and uniform conclusions in liver transplantation, the typical surgery with severe intraoperative hemodynamics fluctuations, regarding the agreement and trending ability of PiCCO to follow up with PAC in terms of hemodynamic monitoring are still lacking.

Our study assessed the agreement and tracking ability by using PiCCO compared to PAC. Our results indicated that, when assessing each parameter in conjunction with specific statistical methods, PiCCO can not be considered comparable to PAC during OLT. These results are specified through two aspects: (1) The Bland-Altman analysis showed that percentage errors for each parameter were outside the acceptable range, indicating poor agreement. Evaluation via ICC and PBR analyses reveals a degree of alignment between the two methods. These analyses consider both systematic and random errors. Additionally, judgments are formed by combining polar plots, four-quadrant plots, and the TIM with professional significance. (2) The acceptability of agreement rates of polar plots, four-quadrant plots and TIM is limited. PiCCO displays a greater negative polarity deviation for each parameter than the recommended ± 5°, and the agreement rate was lower than the recommended 90% [22]. Thus, PiCCO demonstrates a tendency to underestimate the continuous fluctuations during liver transplantation compared to PAC. In terms of tracking consistency trends, inadequate agreement is evident across all parameters.

In addition, our study cohort includes 52 liver transplantation patients from our database, encompassing 18 classical procedure cases, 8 piggyback procedure and 26 ischemia-free procedure cases. The “ischemia-free” technique significantly reduces the incidence of reperfusion syndrome(Post-reperfusion syndrome occurred in three recipients (9%) randomized to ischemia-free liver transplantation (IFLT) and in 21 (64%) randomized toconventional liver transplantation (CLT)(*p* < .001) [35]. IFLT stabilizes the intraoperative hemodynamic, and the patient’s perioperative survival rate is increased by nearly 10%, and the incidence of early liver insufficiency is reduced from 25% to less than 5% [36]. Considering the possible impact of different surgical techniques on our research results, we further compared the"ischemia-free” technique with the conventional liver transplantation technique and founded that there were still significant differences in agreement or trending ability between PiCCO and PAC (Supplementary Material).

PiCCO obtains hemodynamic parameters of patients based on two principles. One is by actual transpulmonary thermodilution, and the other is by arterial pulse wave analysis. The transpulmonary thermodilution method is a non-continuous measurement that can only be obtained at the time of thermodilution for a specific data. In our study, the transpulmonary thermodilution method was mainly used for PiCCO calibration, whereas the continuously obtained CO, CI, SVI and SVRI were all based on the arterial pulse contour analysis. We focused on the arterial pulse wave analysis (pulse contour) by using PiCCO during OLT in our study. PiCCO has good reproducibility, due to the longer transport time of the thermal bolus (20 s), which reduces respiratory-generated artifacts compared with PAC (3–4 s) [37]. In our study, the data from PAC and PICCO at the same time point were compared, but did not consider the influence of respiratory cycles, which maybe one of the reasons for the inconsistency between PiCCO and PAC.

Recalibrated frequency maybe the another reasons for the inconsistency between PiCCO and PAC. The current PAC can automatic calibration continually during surgery. However, continuous measurements of PiCCO are based on the arterial pulse wave analysis, which still requires intermittent transpulmonary thermodilution for calibration; however, transpulmonary thermodilution of PiCCO requires recalibration after significant hemodynamic changes [38, 39]. We routinely recalibrate at the beginning of prehepatic, hepatic-free, and neohepatic phases respectively, or the time after dramatic hemodynamic fluctuations during the operation, but the dramatic hemodynamic fluctuations is not be defined. At the same time, it is difficult to achieve recalibration of PiCCO frequently during the operation. We agree that agreement between the pulse wave assessment by PiCCO and the PAC thermodilution can vary. This latter variation most likely results from “drift” of the arterial-based system, requiring re-calibration. In addition, the choice of correction frequency is important in hemodynamically unstable patients, such as the liver-free and neohepatic phases in our study. We therefore recommend shortening the intraoperative calibration frequency of the PiCCO thermodilution method in liver transplant patients.

The main limitation of our study is that when taking the data from retrospective database, although we try to minimize errors using statistical methods, there are still a lot of confounding factors that influence the measurements and can reflect on results, including technical mistakes, delay between the measurements, etc.

In summary, our study assessed the agreement and tracking ability by using PiCCO compared to PAC during OLT. Our results indicated that, when assessing each parameter in conjunction with specific statistical methods, PiCCO cannot currently be considered comparable to PAC. Actually, PiCCO has been widely used in procedures with severe intraoperative hemodynamics fluctuations such as liver transplantation because of the advantages of being minimally invasive, safe, and having unique measurement parameters. We suggest that the selection of hemodynamic monitoring techniques in liver transplantation should take into account the patient’s physiological condition, surgical technique, and anesthesiologist’s level of expertise. In addition, the clinical use of PiCCO in liver transplantation should consider the advantages of its minimally invasiveness and multiple parameters with the disadvantages of its lack of accuracy. And it may be necessary to shorten the interval of calibration time and recalibrate more frequently during the operation with severe hemodynamics.

## Conclusions

In conclusion, our study shows that PiCCO and PAC are less consistent in measuring CO, CI, SVRI and SVI during OLT. We recommend that full consideration should be given to the PiCCO’s advantages and disadvantages before using alone in liver transplantation, such as the fact that it is a minimally invasive method with multiple parameters but limited correlation with the PAC.

### Electronic supplementary material

Below is the link to the electronic supplementary material.

Supplementary Material 1

## Data Availability

All data generated or analyzed during this study are included in this published article. The data that support the findings of this study are available from the corresponding author upon reasonable request.

## Abbreviations

OLT: Orthotopic liver transplantation
PiCCO: Pulse indicator continuous cardiac output
PAC: Pulmonary artery catheterization
SVRI: Systemic vascular resistance index
CO: Cardiac output
CI: Cardiac index
SVI: Stroke volume index
ICC: Intra-class correlation coefficient
TIM: Trend interchangeability method
PetCO2: End-expiratory carbon dioxide
TEG: Thromboelastography
PBR: Passing-Bablok regression
PE: Percentage error
IFLT: Ischemia-free liver transplantation
