# Supplementary material for: A comparison of hemodynamic measurement methods during orthotopic liver transplantation: evaluating agreement and trending ability of PiCCO versus pulmonary artery catheter techniques
Source: BMC Anesthesiol. 2024 Jun 6;24:201. doi: 10.1186/s12871-024-02582-x (PMC11155023; doi:10.1186/s12871-024-02582-x)
Supplement: Supplementary file 1 — Supplementary Material 1 [file 12871_2024_2582_MOESM1_ESM.docx]

**A Comparison of Hemodynamic Measurement Methods during Orthotopic Liver Transplantation: Evaluating Agreement and Trending Ability of PiCCO versus Pulmonary Artery Catheter Techniques**

Table of contents

Supplementary information.............................................................................2

Figure S1.........................................................................................................3

Figure S2.........................................................................................................4

Figure S3.........................................................................................................5

Figure S4.........................................................................................................6

Figure S5.........................................................................................................9

Table S1.........................................................................................................10

**Supplementary appendix.**

Our study cohort includes 52 liver transplantation patients from our database, encompassing 18 classical procedure cases, 8 piggyback procedure and 26 ischemia-free procedure cases. The "ischemia-free" technique had been shown successfully addresses prolonged "no blood supply" to the liver during transplantation. When the donor liver is implanted into the transplant recipient, the recipient's blood vessels are connected to the Multi-Organ Functional Repair System, and the recipient's blood circulation system "takes over" to complete the liver transplant. During the whole process, the blood flow in the liver does not stop, and the patient's perioperative survival rate is increased by nearly 10%, and the incidence of early liver insufficiency is reduced from 25% to less than 5%(29). Our further comparative analysis of ischemia-free surgical cases showed similar differences in consistency or ability to trend between PiCCO and PAC, as analyzed below.

**Figure S1. Agreement was assessed using Bland–Altman analysis in ischemia-free procedure cases**

**
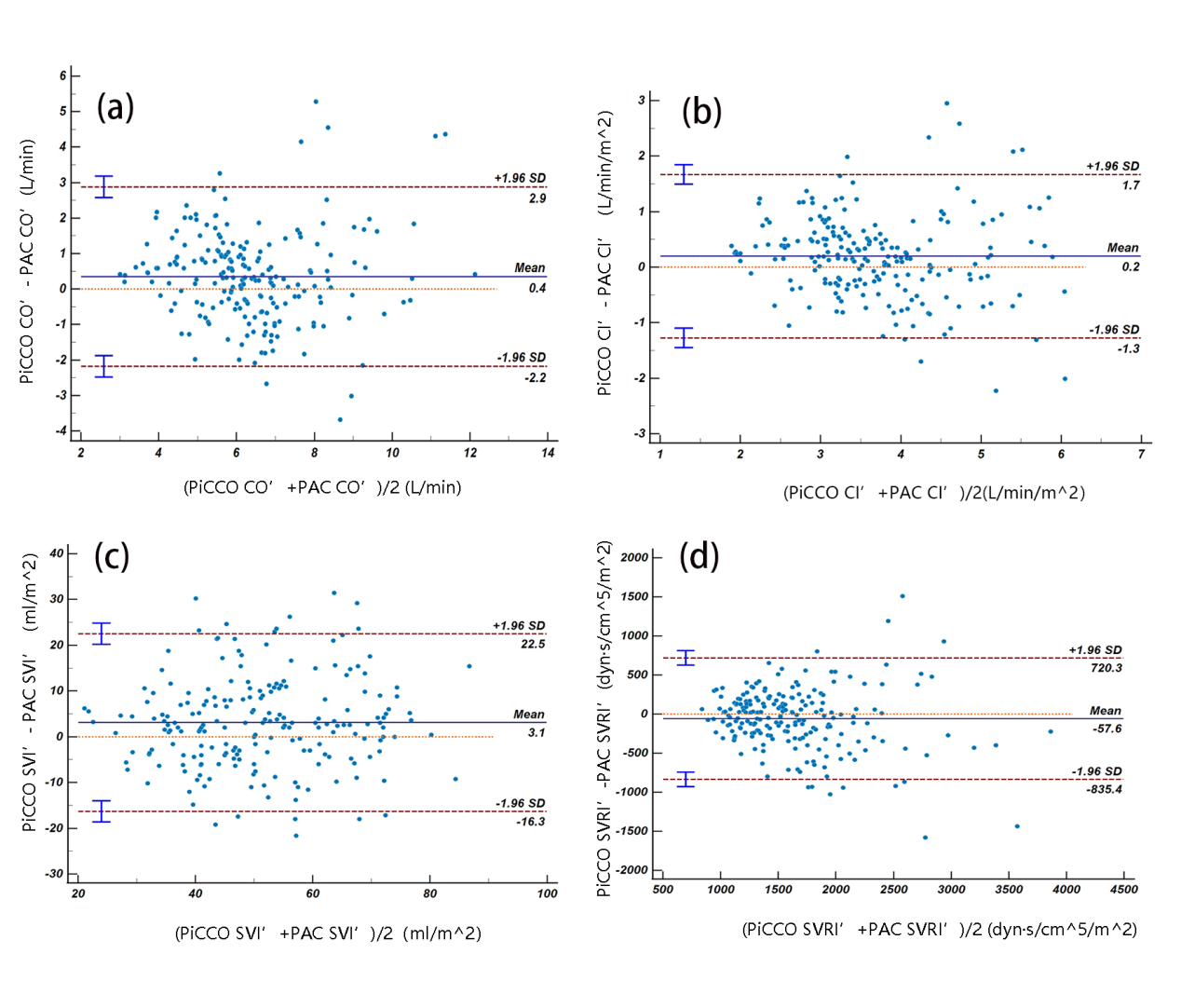
**

The Bland-Altman analyses for ischemia-free procedure cases are shown in Figure S1. (a) Bland–Altman analysis comparing the CO measured using PiCCO with that using PAC. (b) Bland–Altman analysis comparing the CI measured using PiCCO with that using PAC. (c) Bland–Altman analysis comparing the SVI measured using PiCCO with that using PAC. (d) Bland–Altman analysis comparing the SVRI measured using PiCCO with that using PAC. The blue line indicates the mean bias, and the dashed lines indicate the 95% limits of agreement in each analysis. SD, standard deviation.

**Figure S2. Agreement was assessed using Passing-Bablok regression (PBR) in ischemia-free procedure cases**


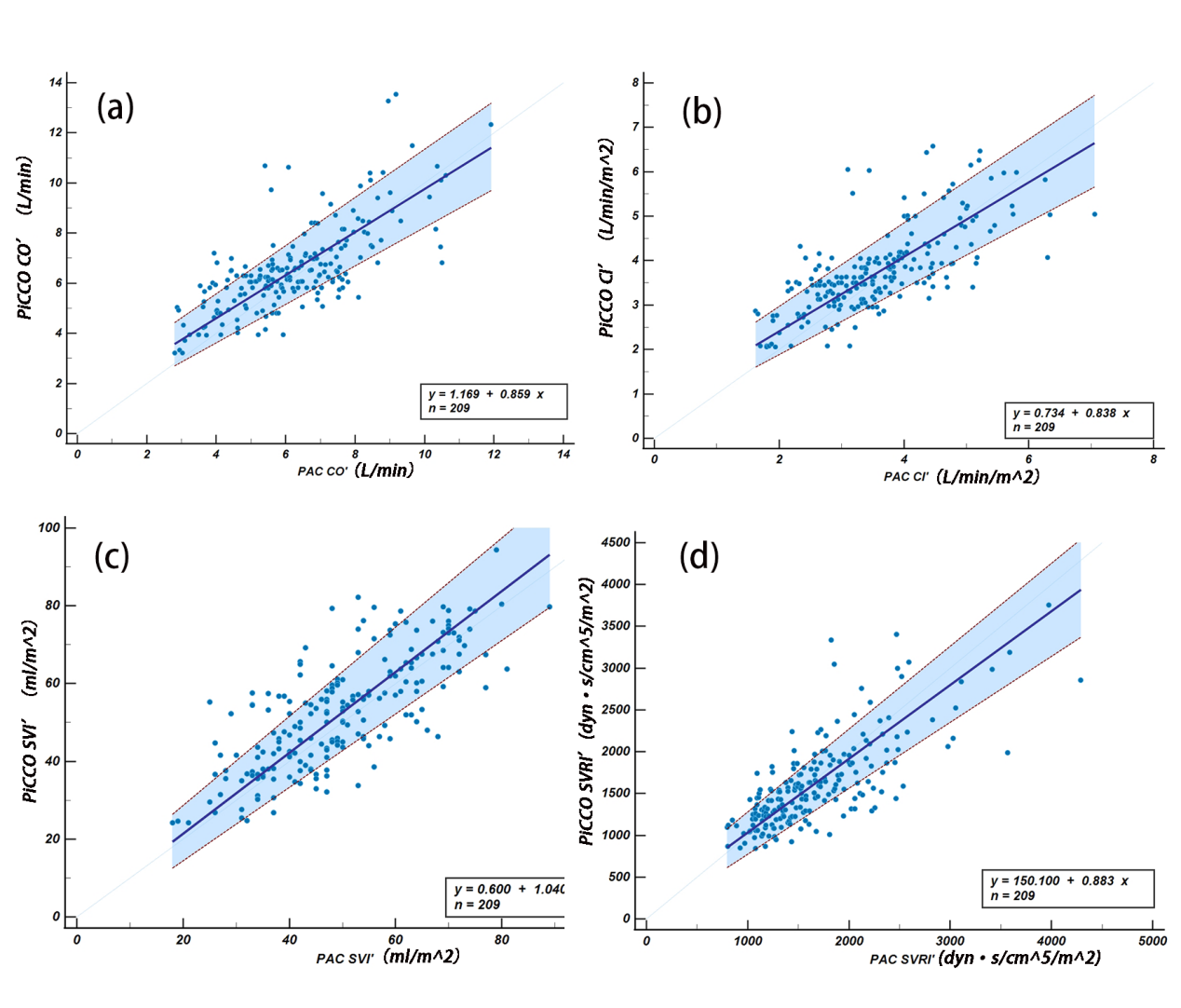


Figure S2 shows the passing-bablok plots for each hemodynamic parameter for two devices. Passing-bablok regression between PiCCO and PAC for CO (a), for CI (b), for SVI (c), for SVRI (d). CO, cardiac output; CI, cardiac index; SVRI, systemic vascular resistance index; SVI, [stroke volume index](http://www.baidu.com/link?url=a_XZXcCgk84_cFNSC-eNI9jJar0wnJ9Y2XIwDApxujhzf2aH5_vH49svvJy_UCmvyhKN1ueAjXsDXIMoLu5dKa).

**Figure S3. Trending ability was assessed using the four quadrant plots in ischemia-free procedure cases**


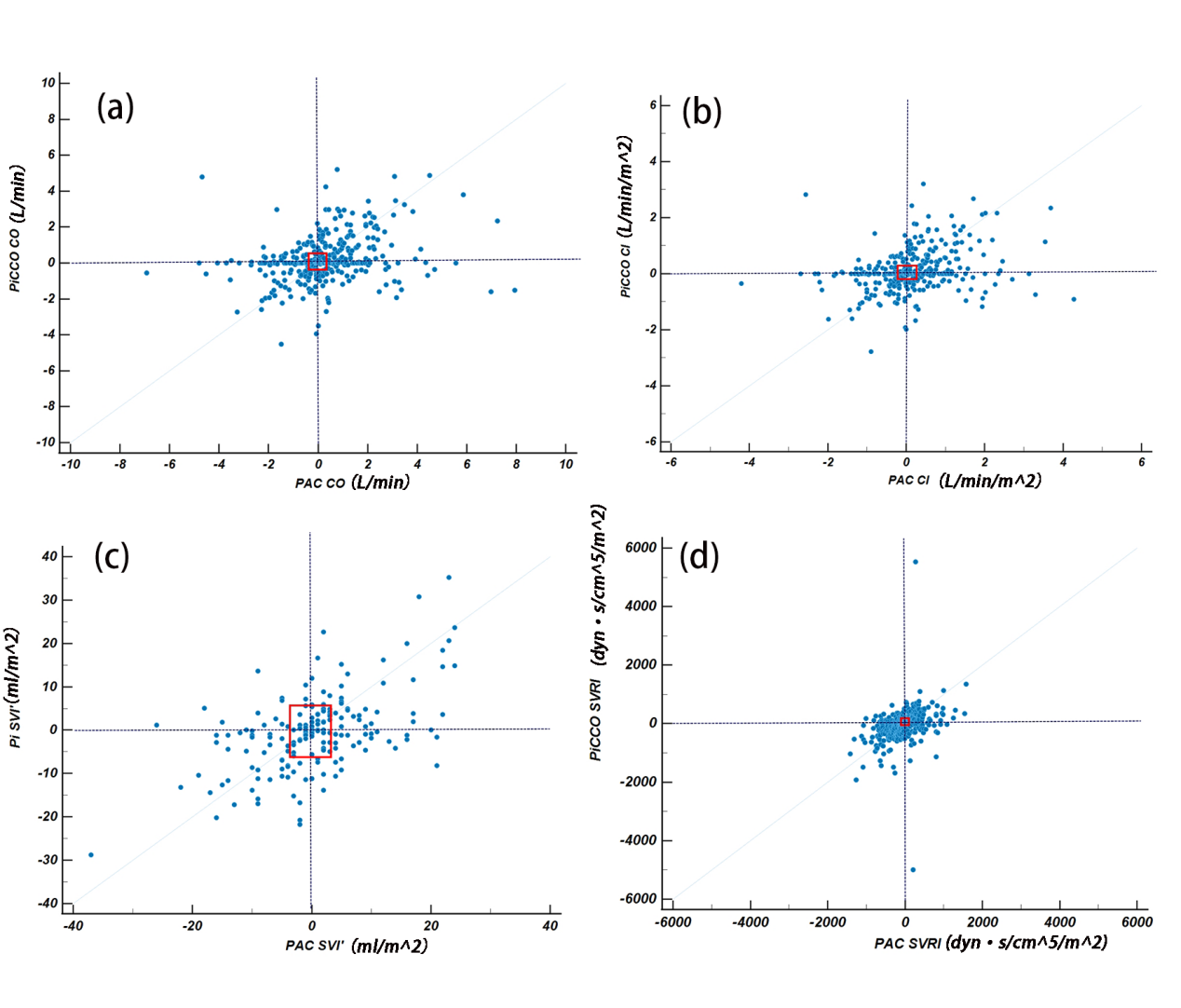


Figure S3. Four-quadrant plot corrected for repeated measurements shows changes in CO(a), CI (b), SVI (c), SVRI (d). The exclusion rates (red squares) for the central region were both set to 10% of the parameter mean.

**Figure S4. Trending ability was assessed using polar plot in ischemia-free procedure cases**


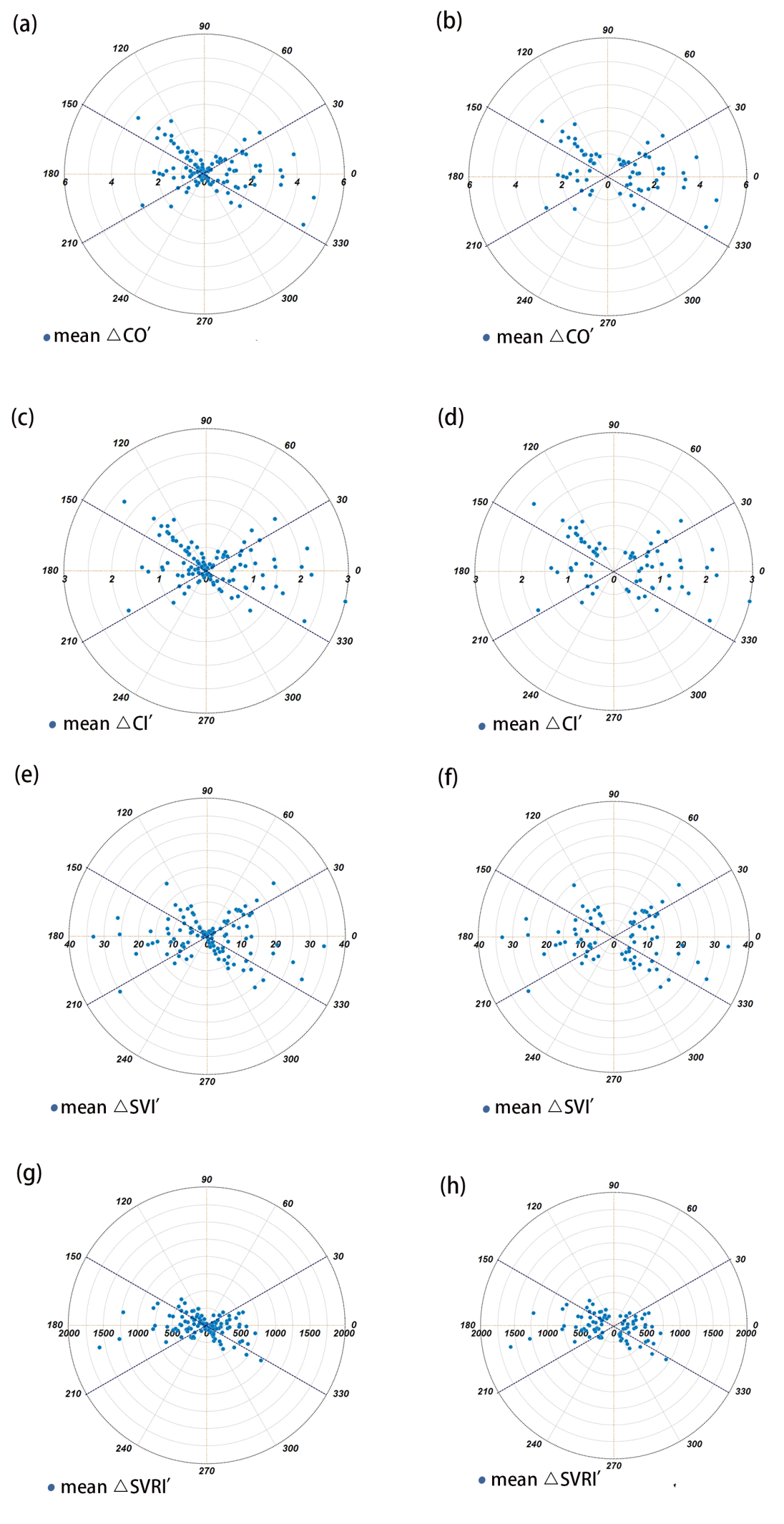


Figure S4. Polar plots used to show trending ability. The distance from the center of the plot represents the mean change in cardiac output (△CO) and the angle with the horizontal (0-degree radial) axis represents agreement (a), the exclusion zones of 10% (b). The distance from the center of the plot represents the mean change in CI (c), the exclusion zones of 10% (d). The distance from the center of the plot represents the mean change in SVI (e), the exclusion zones of 10% (f). The distance from the center of the plot represents the mean change in SVRI (g), the exclusion zones of 10%(h). The radial agreement limit was taken as -30 to +30°, and after excluding data from the central area, a compliance rate above 95% was considered good trend ability, 90% ~ 95% was borderline, and below 90% was poor trend ability.

**Figure S5. Trending ability was assessed using trend interchangeability method（TIM）in ischemia-free procedure cases**


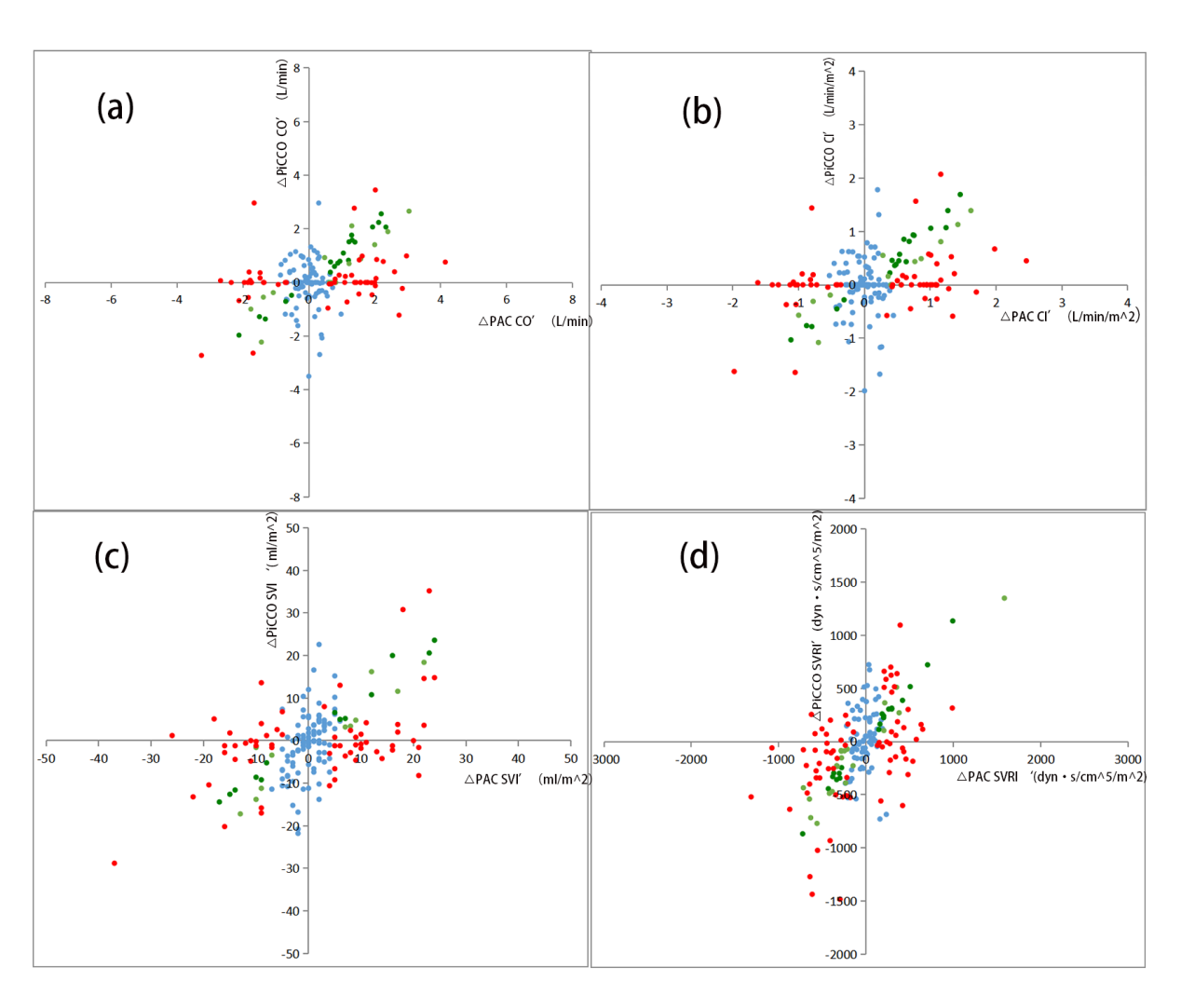


Figure S5. Four-quadrant graphical representation between changes in absolute values of CO measured by PiCCO and PAC ( 364 pairs of data points) according to the trend interchangeability method(TIM). (b) Four-quadrant graphical representation between changes in absolute values of CI measured by PiCCO and PAC according to the TIM. (c) Four-quadrant graphical representation between changes in absolute values of SVI measured by PiCCO and PAC according to the TIM. (d) Four-quadrant graphical representation between changes in absolute values of SVRI measured by PiCCO and PAC according to the TIM. A specific colour is applied to each change: uninterpretable (blue), non-interchangeable (red), in the grey zone of interpretation (orange), and interchangeable (green).

|  | clinically acceptable standard | CO | CI | SVRI | SVI |
| --- | --- | --- | --- | --- | --- |
| Concordance rate of 4-quadrant plot(n%) | ≥90 | 73 | 73 | 75 | 72 |
| Angular deviation of polar plot(n°) | -5~5 | 68 | 69 | 78 | 77 |
| Concordance rate of polar plot(n%) | ≥90 | 52 | 54 | 73 | 61 |
| The interchangeability rate of TIM(n%) | ≥90 | 23 | 23 | 18 | 16 |

Table S1. The consistency rates of the three methods of tracking trends in ischemia-free procedure cases. These results suggest that there are still differences between PiCCO and PAC in terms of agreement and trending ability.
